# Personalized Medicine in Malignant Melanoma: Towards Patient Tailored Treatment

**DOI:** 10.3389/fonc.2018.00202

**Published:** 2018-06-12

**Authors:** Hildur Helgadottir, Iara Rocha Trocoli Drakensjö, Ada Girnita

**Affiliations:** ^1^Skin Tumor Center, Theme Cancer, Karolinska University Hospital, Stockholm, Sweden; ^2^Cancer Centrum Karolinska, Karolinska Institutet Stockholm, Stockholm, Sweden

**Keywords:** melanoma, receptor tyrosine kinases, signaling, ubiquitination, targeted therapy, functional selectivity

## Abstract

Despite enormous international efforts, skin melanoma is still a major clinical challenge. Melanoma takes a top place among the most common cancer types and it has one of the most rapidly increasing incidences in many countries around the world. Until recent years, there have been limited options for effective systemic treatment of disseminated melanoma. However, lately, we have experienced a rapid advancement in the understanding of the biology and molecular background of the disease. This has led to new molecular classifications and the development of more effective targeted therapies adapted to distinct melanoma subtypes. Not only are these treatments more effective but they can be rationally prescribed to the patients standing to benefit. As such, melanoma management has now become one of the most developed for personalized medicine. The aim of the present paper is to summarize the current knowledge on melanoma molecular classification, predictive markers, combination therapies, as well as emerging new treatments.

## Background

Cutaneous melanoma (CM), a malignant tumor derived from melanocytes, is the deadliest form of skin cancer. Risk factors for melanoma include family history, sex, age, skin pigmentation, sunburn susceptibility, tanning ability, nevus count, freckling, and psychological health. In addition, socioeconomic features, in particular, the occupation, access to health and prevention measures, latitude, or level of ozone layer have been demonstrated to impact the morbidity of melanoma (1). Nevertheless, exposure to ultraviolet radiation (UVR) remains the most important causative factor of melanoma development (2). Notably, dose intensity within the total amount of UVR is of paramount importance (3). Thus, large intermittent UVR doses are associated with a higher risk of developing melanoma when compared to the same amount of chronic UVR received in smaller doses (2).

Melanoma incidence is among the most rapidly increasing cancers within Caucasian populations worldwide (4). In Sweden, the age-related incidence per 100,000 inhabitants between the years 1970 and 2015 rose from 6.3 to 35 in men and from 7.7 to 32.6 in women (5). The melanoma mortality (cause of death by melanoma per 100,000 individuals) in Sweden increased from 4.1 in 1997 to 5.6 in 2016, and this trend is predicted to continue (5, 6).

Despite a significant improvement in treatment during the last couple of years, metastatic melanoma continues to be a major clinical challenge. Consequently, there is a need for further research on disease etiology and pathogenesis leading to the identification and validation of drug targets and biomarkers. Such efforts will hopefully lead to an improvement in preventive measures, early diagnosis, and personalized treatments.

Personalized medicine or precision medicine is a medical procedure that separates patients with the same diagnosis into different groups—with interventions, treatments, or other medical decisions, specifically designed for the individual patient, based on their predicted response or risk of disease (7). The concept is based on adequate knowledge of the molecular and cellular mechanisms of the disease as well as availability of appropriate investigations and treatments. Implementation of such a model for melanoma requires an update of its classification system. The aim of the present paper is to summarize the current knowledge on melanoma classification, predictive markers, combination therapies, as well as emerging new treatments.

## Melanoma—Classical System

Cutaneous melanoma is divided into different clinical types. The most common ones include superficial spreading melanoma (SSM), nodular melanoma, lentigno maligna melanoma (LMM), and acral lentigious melanoma (ALM). Less common forms are spitzoid, desmoplastic, or nevoid melanoma. This classification defines only the clinical appearance without providing any information regarding the prognosis. Such information is indicated by the TNM staging system, currently used worldwide and endorsed by the American Joint Committee on Cancer (AJCC). This takes into consideration the tumor size (T), loco-regional dissemination to the lymph nodes (N), and occurrence of distant metastasis (M) (8). The staging of primary melanoma is based on tumor thickness (Breslow) as well as tumor ulceration. Thin melanoma (0.1–1 mm in Breslow scale) has a lower risk for metastasis and thus better prognosis compared to thicker melanoma (>1 mm) (9).

Lentigo maligna (LM) is the most common type of melanoma *in situ*, representing about 83% of all *in situ* melanoma (10) and three times more frequent than SSM (11). It affects mostly chronically sun-exposed areas like the head and neck of middle aged and elderly Caucasians (12, 13). The incidence of LM is estimated to be 13.7 per 100,000, and it is expected to rise in most high-income countries (14). A recent study from Netherlands detected a 12.4% increase in incidence between 2007 and 2013 (15). If left untreated, LM can progress to the invasive form of LMM, representing 4–15% of all invasive melanomas (12). The exact rate of change and time for LM to develop into LMM is not known (16, 17). For patients diagnosed with LM at age 45, epidemiological analysis estimated a 5% lifetime risk of developing LMM (16) while the absolute risk of developing a LMM at any location, after a histologically confirmed LM, was shown to be lower (2.0–2.6%) (15). Yet, other studies have reported a higher invasion rate, detecting the presence of invasive foci in 16–50% of all tested LM (18, 19). The invasive form (LMM) has the same prognosis and the potential to metastasize as other forms of melanoma (14, 15, 20).

Acral lentiginous melanoma is a term used to describe melanomas arising from palms, soles, and nail beds. ALM accounts for 2–3% of all CMs and is the most common subtype in patients with skin of color. Mechanical stress and history of trauma are described as risk factors for development of ALM and the association with a preexisting nevus is unusual (21, 22). The ALM survival rate is 10–20%, lower than CM, and this worse prognosis is mainly associated with socioeconomic factors that contribute to delayed diagnosis rather than the natural history (22, 23).

Mucosal melanoma (MM) is the rarest subtype, corresponding to 1.3% of all melanomas. It emerges from melanocytes present in the mucous membranes of the respiratory, gastrointestinal, genitourinary tract, and the eye (sclera and conjunctiva), with only the latter being associated with UV radiation (24–26). Head and neck are the most common location, accounting for more than half of all MM, and involve the nose and paranasal sinuses, oral cavity, pharynx, and larynx. Most studies report a similar distribution of MM between men and women with the notable exception of genital tract melanoma displaying a higher rate in women. The incidence of MM varies also between races, accounting for a greater proportion (8%) of all melanomas in Japanese patients as compared with Caucasians (1%). The hidden location and the rich vascularization of the mucous membrane are factors that contribute to a poorer prognosis when compared to CM, with a 5-year overall survival rate of only 25% (27, 28).

Uveal melanoma (UM), the most common form of ocular melanoma, is a tumor that arises from melanocytes in the eye. UM is as a disease entity very distinct from cutaneous or conjunctival melanoma, with entirely different etiology, epidemiology, biology, genetics, and clinical aspects. The annual incidence of UM is 2–8 cases per million inhabitants, yet unlike CM, there is no increasing trend for UMs incidence over the last decades (29, 30).

## Molecular Classification of Melanoma

Over the last decade, new therapies for melanoma have been developed, with impressive effects on survival. Personalized medicine allows earlier intervention as well as the possibility to choose more efficient therapies tailored to the specific patient. Since classical classification systems are limited in terms of prognosis and prediction of treatment response, a new system for melanoma classification is needed. Such a system will incorporate the biological and molecular aspects of the disease, now recognized.

**Figure 1:**
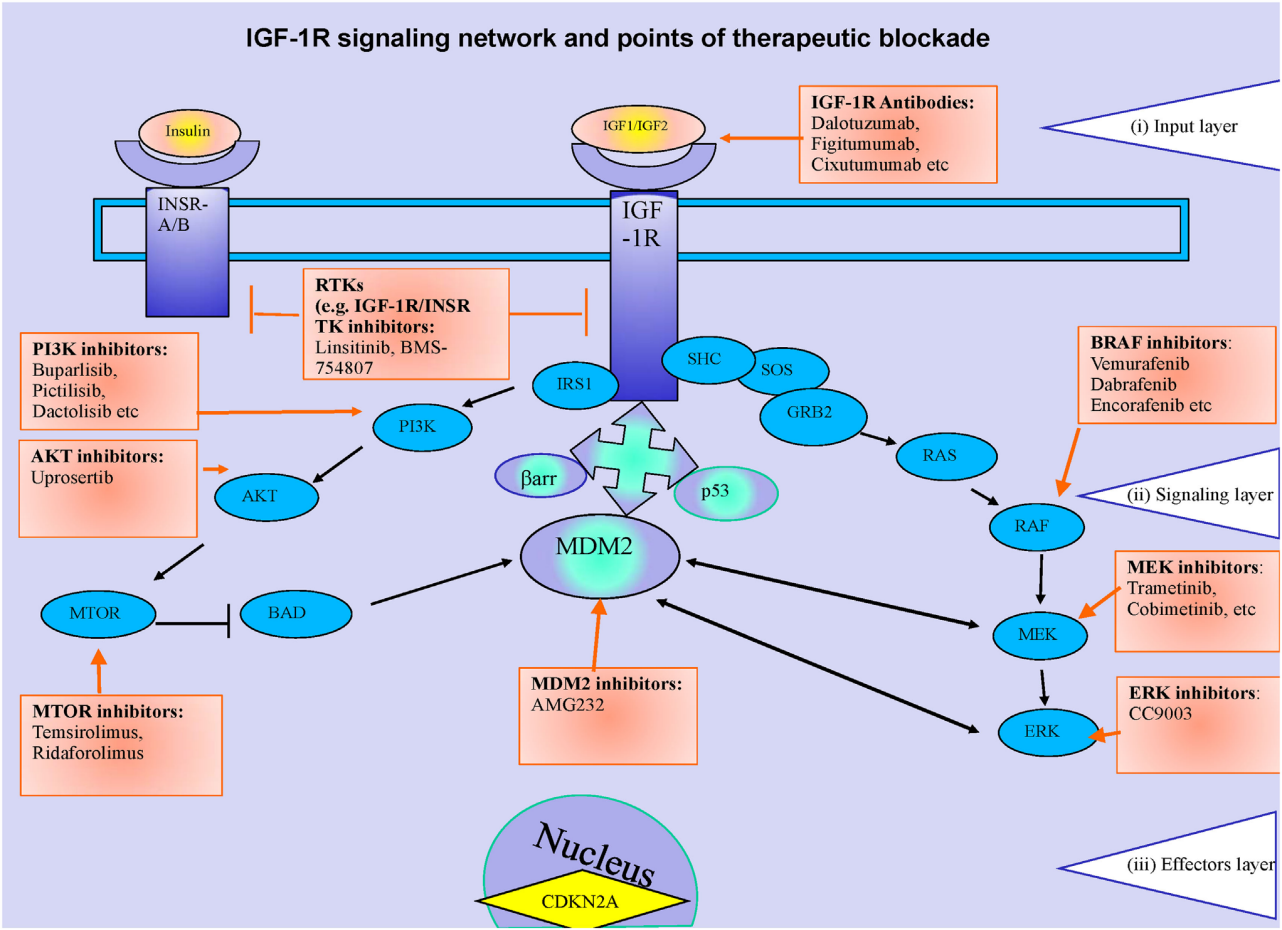
Receptor tyrosine kinases (RTKs) (IGF-1R) signaling network and points of therapeutic blockade. The canonical RTKs signaling can be represented as a system of three layers. The input layer (i) is made up of ligands (e.g., insulin, insulin-like growth factor 1, IGF-2) and surface receptors (IGF-1R, insulin receptor). Ligand–receptor interaction initiates activation of the second layer (ii) (the signaling cascade) through recruitment of the two main adaptor proteins; Shc and the IRS’s. Through stepwise enzymatic activation, the signal cascade is set up, following two main routes—the mitogen-activated protein kinase (MAPK) route and the phosphoinositide 3-kinase (PI3K) route. The signaling cascade arms culminate in the activation of transcription factors in layer (iii), which control site specific transcription and generate the resulting biological effects. The molecular changes associated to melanoma pathogeny and hence potential therapeutic targets are indicated within each of the system’s layers.

Melanomas are known to be very heterogeneous in their nature, reflecting the molecular changes occurring in the development of the disease, and as such there is no “one size fits all” regarding the treatment. In fact, compared to other tumor types, melanoma has an exceptionally high frequency of acquired mutations (31). Some molecular events are more frequent and provide the clinician the opportunity of adjusting the treatment to the individual patient. Depending on the subcellular level, these events can be categorized within a system of three distinct layers (Figure 1). The input layer-plasma membrane (i) is made up of ligands and surface receptors. Upon stimulation, through stepwise enzymatic activation, a signal is transmitted through to the second layer [signaling layer (ii)], following two main routes—the mitogen-activated protein kinase (MAPK) route and the phosphoinositide 3-kinase (PI3K) route. The signaling cascade ends with the activation or inhibition of transcription factors in the effector layer (iii), controlling specific gene transcription and resulting in the specific biological effects.

Within the *input layer*, representative examples for melanoma are the receptor tyrosine kinases (RTK), c-KIT, and anaplastic lymphoma kinase (ALK). C-KIT (CD117) is expressed on a wide variety of cell types and after stimulation by its cognate ligand stem cell factor (SCF), induces activation of PI3K-AKT-mTOR, RAS-RAF-MEK-ERK, and the signal transducer and activator of transcription 3 (STAT3) pathways. C-KIT mutations occur most frequently in acral melanomas (10–20%) and MMs (15–20%) (32, 33). ALK mutations have been reported in spitzoid melanoma and can be associated with concomitant BRAF or NRAS mutation. It is expressed normally in the brain, small intestine, and testis, but not in normal lymphoid cells (34). ALK shows greatest sequence similarity to the insulin receptor subfamily of transmembrane tyrosine kinases.

The *second layer* is probably the site with the highest frequency of mutational events in melanoma ultimately responsible for constitutive activation of signaling pathways through G-protein, phosphatidyl-inositol or kinase-cascades.

Guanine nucleotide-binding proteins (G proteins) are a family of heterotrimeric proteins, which couple receptors for neurotransmitters, growth factors, and hormones to intracellular signaling pathways. For melanoma, point mutations have been described for the genes encoding G(q) alpha subunit proteins, GNAQ, and its paralog GNA11. Mutations in these genes are often seen in UM, both in the primary tumor and metastases; however, they are infrequent (GNAQ) or absent (GNA11) in extraocular melanoma (35).

The PTEN–PI3K–AKT pathway is negatively regulated through PTEN, an inhibitor of the PI3K kinase. Inactivation of PTEN promotes cell survival by downregulating pro-apoptotic signaling downstream of AKT and BAD pathways. The tumor suppressor gene PTEN is the third most frequently mutated gene in melanoma after BRAF and NRAS and promotes cell survival (36). A more detailed description of the mutations within kinase signaling cascades are described in the therapy section.

Within *the third layer*, several “effectors” were identified to be mutated in melanomas. Progressive shortening of telomeres with each cell division is a characteristic of normal aging cells, and may be hastened by exposure to harmful environmental exposures such as UVR or tobacco smoke. Such shortening determines cell fate, so, a key event in acquisition of cellular immortality is the upregulation of a telomere length maintenance mechanism. The enzyme telomerase lengthens telomeres by synthesizing new telomeric DNA to compensate for the replication-associated telomere shortening. Telomerase is a complex molecule with several subunits, including the reverse transcriptase TERT. Mutations in the TERT gene promoter are found in many cancers, including melanoma, where it has been reported that UVR signature mutations are seen in 33% of primary melanomas and in 85% of melanoma metastases (37).

Identification of molecular events driving melanoma progression inspired a molecular classification of melanoma within The Cancer genome atlas network (TCGA) (38). This system, based on the status of three genes frequently mutated in melanoma—BRAF, RAS, and NF1—categorized melanoma into different classes, with broad therapeutic implications (38):

- Class 1 (with clinically actionable alterations):
  - BRAF, CDK, MDM2/p53 interaction and PI3K7 AKT/mTor inhibitors for BRAF positive tumors.
  - MEK, CDK, PI3K7 AKT/mTor inhibitors for RAS positive tumors.
  - PI3K7 AKT/mTor inhibitors for NF1 positive melanomas.
  - C-Kit, PKC, CDK, MDM2/p53 interaction and PI3K7 AKT/mTor inhibitors for triple negative.
- Class 2 (with translationally actionable alterations that still require additional evidence to support use in point-of-care decision making):
  - ERK, IDH1, and (PPP6C) Aurora kinase inhibitors for BRAF and RAS positive melanoma,
  - MEK, ERK, and IDH1 inhibitors for NF1,
  - IDH1 for the triple negative tumors.
- Class 3 (with preclinical evidence that has demonstrated biological importance but has not yet demonstrated clinical relevance) biomarkers like ARID2 chromatin remodelers (synthetic lethality) for the three types of melanoma and (BCL2) BH3 mimetics for triple negative.

Last but not least important, immune infiltration is statistically correlated with more favorable prognosis, irrespective of genomic subtype and as such the use of immunotherapy strategies would be included in class 1 (clinically actionable alteration).

## Melanoma Therapy, Today and Tomorrow

### New Melanoma Therapies in Current Clinical Practice

For the primary melanoma, surgery is still the gold standard, with low risk of dissemination and good results for long term survival, mainly for thin melanomas. For LM radiation therapy (RT), in particular, Grenz rays (GR) treatment (soft rays) has proven to be a non-invasive treatment with clearance rates comparable to those of conservative surgery (39). Grenz rays are very superficial RT (typically 10–20 kV), which only penetrate to the epidermal–dermal interface (no deeper than 2 mm) and has been used as definitive treatment or as adjuvant treatment on finding positive margins following excision (39, 40). The doses that penetrate the skin exponentially falls with depth with GR and hence is not recommended for treating LM deeper than 0.8 mm (40, 41). In order to reduce tissue necrosis, the total dose, which ranges from 100 to 160 GY is fractionated into six sessions (41). The treatment is usually painless and well tolerated by patients (41). A margin of 1 cm is usually applied to reduce risk of recurrence. A study of 593 patients treated with GR with a follow-up of between 2 and 5 years, presented an overall curative rate of 88% (41). A follow-up study investigating the efficacy of initial treatment of LM with surgical excision (42) showed good efficacy of GR while the most common side effects were acute dermatitis and necrosis shortly after treatment. Hyper and Hypopigmentation, skin atrophy, and telangiectasia were also observed and usually appear 6 months after the radiation (41). Another drawback is the inconvenience for frail patients to attend the hospital visits during the treatment (43).

On the other side of the spectrum, disseminated melanoma has until recent years been considered one of the more therapy-resistant malignancies. Since dacarbazine was FDA approved in the 1970s, no other chemotherapy agents had been accepted for treatment of metastatic melanoma (44) until two new main groups of drugs got the green light for clinical use: inhibitors of signaling of Ras–Raf–MEK–ERK pathway downstream different RTK (IGF-1R, KIT) and the immune checkpoint inhibitors.

#### Ras–Raf–MEK–ERK Pathway

The Human Genome project significantly contributed to the ability to perform large-scale DNA studies (20, 45). One of the many results from this was the identification of high-frequency mutations in the BRAF and NRAS oncogenes. This has subsequently led to the discovery of selective inhibitors targeting the Ras–Raf–MEK–ERK pathway that is often constitutionally activated in melanoma (46). Activating BRAF mutations at codon 600 (mainly V600E) are found in approximately 50% of melanomas, particularly, in nodular and SSMs (46). Vemurafenib was the first FDA approved BRAF-inhibitory therapy for BRAF mutated melanoma. Objective, rapid, and sometimes striking complete responses (CRs) are seen in around 50% of metastatic melanomas, but therapy resistance and relapse usually develops after some months. Although the tablet-form therapy is generally well tolerated, it is sometimes complicated by side effects such as photosensitivity and fast evolving benign or sometimes malignant keratinocytic skin lesions (47). Later, the BRAF inhibitor dabrafenib was approved. Dabrafenib has similar antitumor effect as vemurafenib and is not associated with the same photosensitivity, but instead pyrexia is more frequent (48). Addition of the MEK-1 inhibitors such as trametinib and cobimetinib has shown further improvements in survival, and fewer incidences of keratinocytic lesions, although pyrexia is more frequent. Currently, combination therapy with dual inhibition of BRAF and MEK is considered standard treatment for BRAF mutated melanoma (49).

Imatinib mesylate (Gleevec), an oral KIT inhibitor has shown some short lived effect in KIT mutated MM but no efficacy in UM (50–52). Some ALK inhibitors (crizotinib, ceritinib, and alectinib) are now approved for clinical use in lung cancer, but not yet in melanoma (53).

#### Immunotherapy

The long journey of melanoma immunotherapy started with important recognition of lymphocytes specifically activated by melanoma antigens (54). This marked the beginning of an extended quest to identify immune-based antitumor regimens, leading to the FDA approval of adjuvant therapy in stage III melanoma with high-dose interferon in 1996 and of high-dose bolus IL-2 for advanced melanoma in 1998. Due to relative low response rates and considerable side effects, these drugs are generally not recommended for melanoma treatment. However, these translational discoveries within tumor immunology paved the way for subsequent discovery of targeted immune modulating therapies that, compared to standard chemotherapy, showed superior outcomes in randomized studies (55). The first approved checkpoint inhibitor was ipilimumab with a response rate of about 15%, but sometimes-remarkable durable remissions (56). The treatment has substantial, immune related, even life-threatening side effects such as colitis, hepatitis, dermatitis, and hypophysitis. The PD-1 blocking immune checkpoint inhibitors pembrolizumab and nivolumab were later shown to have higher response rates, improved progression-free and overall survival, and milder side effects than ipilimumab (57, 58).

While targeted therapies have revolutionized the advanced melanoma treatment, some patients are not responsive due to preexistent (giving an intrinsic) resistance or the clinical responses are mitigated by mechanisms activated by drug administration (acquired resistance). The intrinsic resistance is especially relevant for vemurafenib or dabrafenib therapy, with about 20% of patients with BRAF-mutated melanoma not-responsive (59). This is not surprising as melanoma displays a broad degree of genomic lesions and tumor heterogeneity and hence a diverse spectrum of resistance mechanisms. Likewise, acquired resistance to drugs targeting RAF pathways develops in about 50% of responders 1 year after initiation of therapy and involved two main settings (59): (i) alternate activation of other components of the RAS/RAF/MEK/ERK (e.g., activating mutations in genes encoding MEK1, MEK2, or RAS proteins) or (ii) activations of the mechanisms initiating RAS/RAF pathways such as enhancement of RTKs signaling [e.g., platelet-derived growth factor receptor (PDGFR), MET, IGF-1R], hyper activation of the parallel PI3K–AKT pathway through PTEN mutations or upregulation of the transcription factors (e.g., STAT3, PAX3) (60). On the other hand, for the therapeutic strategies involving T cells checkpoints inhibitors, (e.g., PD-1), about 50% of the patients will initially respond, yet, these responses have a tendency to last longer (61). It is beyond the scope of the present review to discuss in details the mechanisms for intrinsic and acquired resistance to targeted therapy in melanoma and the reader is referred to recent comprehensive reviews (59–61).

## Predictive Markers

Several clinical markers are used to predict responses to both immunotherapy and BRAF/MEK inhibitors. High lactate dehydrogenase levels, worse performance status, high tumor burden, more metastases sites, and brain metastasis are all clinical parameters that are associated with worse therapy responses. Recently, more advanced predictive methods are being developed including the use of liquid biopsies and patient-derived xenografts (PDX) described below.

One of the most frequently cited reasons for the high failure rate of new agents in melanoma treatment are the lack of preclinical models that recapitulate the heterogeneity of tumors in patients. PDX or the development of “Mouse Avatars” (62) entails implantation of patient tumor samples in mice for subsequent use in drug efficacy studies. These avatars allow for each patient to have their own tumor growing in an *in vivo* system, thereby allowing the identification of a personalized therapeutic regimen, eliminating the cost and toxicity associated with non-targeted chemotherapeutic measures (63). The approach is very straightforward, consisting of obtaining fresh surgical tissue, sectioning it into ~3 mm^3^ pieces, followed by subcutaneous or orthotopic implantation into the flank of an immune deficient mouse or rat. One of the main advantages of PDX models is maintenance of the original tumor architecture and histology (63) characteristics. A drawback to this model is that it is not feasible to study efficacy of immune therapies in immune deficient animals, but this has recently been compensated for in a mouse model where patient T-cells are transplanted parallel to tumor tissue (64).

In the last two decades, advances in the detection and characterization of circulating tumor cells (CTCs) and cell-free circulating tumor DNA (ctDNA) in plasma and serum of cancer patients, including those with advanced melanoma, has revolutionized the field of genomic biomarker assessment. As a non-invasive test, the diagnostic and prognostic potential of CTC and ctDNA by liquid biopsy provides actionable molecular information previously only available from tumor tissue (65).

Expression of PDL-1 has been also shown to be a reliable indicator of response to anti-PD-1 therapy. Combination therapy with PD-1 inhibitor nivolumab and CTLA-4 inhibitor ipilimumab further improves progression-free survival (PFS) in patients with metastatic melanoma; however, the combination therapy increases the risk of immune-related adverse events (58). Patients who have melanoma tumors with a low expression of programmed death-ligand 1 (PD-L1) have been shown to benefit more from this combination therapy. PD-L1 thus serves as a predictive marker in the selection of single agent or combination immunotherapy in melanoma patients.

The immune checkpoint therapies appear to have superior effects in melanoma compared to many other tumor forms. This is probably a result of the innate immunogenicity of melanomas tumors with their higher mutation burdens compared to other types of tumors. Mutation load is a marker that, in the future, will likely be incorporated in the clinic as a predictive marker for immunotherapy. Additionally, recent studies have shown that the novel melanoma agents not only have a role in the treatment of disseminated melanoma but also prolong survival in the adjuvant situation. It is thus anticipated that both combination therapies with BRAF/MEK inhibitors and checkpoint inhibitors will be approved in patients who have had high-risk melanomas resected (66).

### Near Future Therapies

#### Potential Drug Targets in Melanoma

As most of the available therapeutic strategies aim to mitigate signaling activation, its transduction or their ultimate transcriptional effects, an improved classification system might be based upon the subcellular level of drug target. Furthermore, the drugs might target directly the malignant cells or indirectly, through activation of the host immune system (Table 1).

**Table 1:** Established and potential targets for the treatment of melanoma and their level of action.

Type of target	Examples of drugs/agents	Comment	Selected Reference
**Established effective targets on plasma membrane**			
c-kit	Imatinib (Glivec, Imatinib)	In mucosal melanoma	(50, 146)
IGF-1	Linsitinib	Phase I i association with Erlotinib	(147)
Epidermal growth factor	Gefitinib (Iressa), Erlotinib (Tarceva)	Approved for lung cancer, studied on melanoma, both cutaneous and uveal	(148, 149)

**Potential effective targets on plasma membrane**			
GNAQ/GNA11	PKC inhibitor AEB071 (sotrastaurin)	In uveal melanoma	(150)

**Established effective targets within signaling transduction pathways**			
BRAF	Vemurafenib, dabrafenid, encorafenib	In skin, melanoma binds to and inhibits activated BRAF	(47, 48)
MEK	Trametinib, cobimetinib, binimetinib	Often associated with BRAF inhibitors to overcome acquired resistance	(49)

**Potential effective targets within signaling transduction pathways**			
NRAS	Farnesyltransferaze inhibitors (R115777)	Most frequently mutated at hotspots in exon 1 (codon 12) and exon 2 (codon 61), which results in the prolongation of its active GTP-bound state	(91, 151)
PI3K	Pictilisib	In melanomas with *PTEN* aberrations	(96)
ALK	Crizotinib	In uveal and spitzoid melanoma	(152)
CDK4/6	Abemaciclib, palbociclib	In melanomas with *CDKN2A* aberrations	(99, 100)

**Established effective nuclear targets**			
None described so far			

**Potential effective nuclear targets**			
MITF	CH5552074	Inhibition of cell growth by reducing the expression level of MITF protein	(153)
TERT	*In vitro* test with Telomerase inhibitor IX	Acral and cutaneous melanoma	(154)
BAP1	*In vitro* with ubiquitin vinyl sulfone (Ub-VS)	In uveal melanoma	(105)
Histone deacetylases	Entinostat	In association with pembrolizumab in melanoma	(109)
			(159)

**Established effective immune targets**			
CTLA-4	Ipilimumab	T-cell activator and blocks B7-1 and B7-2 T-cell co-stimulatory pathways	(155)
PD-1	Pembrolizumab, nivolumab	Binds to PD-1 and as such activates T-cell-mediated immune responses	(57, 58)
PDL-1	Atezolizumab		(156)
IDO	Epacadostat		(157)

**Potential effective immune targets**			
SD-101		*Via* the toll-like receptor 9	(127)
OX40		Co-stimulatory molecule that can be expressed by activated immune cells	(127)
CD137		Member of the TNFR super family	(127, 158)
GITR		Glucocorticoid induced TNF receptor	(127)

##### Drugs Targeting the Input Layer-Plasma Membrane Receptors

Cell surface receptors (membrane receptors, transmembrane receptors) are modified proteins, embedded in the plasma membrane, which allow communication between the cell and the extracellular space. They are specialized in receiving information *via* extracellular molecules and transmitting them intracellularly to be converted into biological responses. Members of the RTK family have long been demonstrated to control critical steps in melanoma initiation, progression, and metastasis. Few preclinical studies employing overexpression of specific RTKs or increased kinase activity mostly through activating mutations in melanoma have proved the concept of RTKs controlling the aggressive malignant melanoma phenotype as well as resistance to therapy (67).

Mutations of the main RTKs transducer—RAS/BRAF—are events more frequent than mutations in RTKs, yet, mutated forms of RTK genes including KIT, ERBB1-4, PDGFR, the EPH and FGFR families, and others are known to govern abnormal signaling driving aberrant growth and survival of melanoma cells (68).

Among the RTK signaling activated through mutation, one of the best examples is probably represented by c-Kit. Like other RTKs expressed by normal melanocytes, c-kit is encoded by proto-oncogenes (69) and its expression has been documented in a wide variety of human malignancies. However, it is the c-kit kinase activity that has been implicated in the pathophysiology of a number of these tumors, including GIST, neuroblastoma, and CM (50). In CM, c-kit has been shown to be expressed in epidermal melanocytes but is lost in cells infiltrating the dermis (70). Even if c-kit seems to be downregulated during the development of normal melanocytes to melanoma with metastasizing potential, there is no evidence that c-kit-negative cells feature mutations in the *KIT* gene or in its promoter (71–73). In contrast, among tumors expressing c-kit, gain-of-function mutations have been found in mastocytosis (74), seminomas (75), and in GISTs (76). Because all these neoplasms arise in tissues with development that is dependent on the activity of the SCF/c-kit axis, aberrant activation of this axis may be of pathogenic relevance. Data from studies on GISTs showing a substantial proportion contain a mutation in exon 11 leading to ligand-independent c-kit activation, have shown that treatment with STI571, an inhibitor of PDGFR, Bcr-Abl, and c-kit tyrosine phosphorylation, causes tumor regression (77).

Kinases are largely recognized as “drugable” targets, supporting the rationale for characterization of RTKs activity in melanoma. Yet, the clinical success of nearly all tyrosine-kinase inhibitors is predicted by the presence of activating mutations or substantial receptor overexpression as is the case with all RTKs presented in the section. On the other hand, this is not the case for another prominent member of the RTKs family, the insulin-like growth factor receptor (IGF-1R). Large amounts of preclinical, epidemiological, and clinical data clearly demonstrate a role for the IGF-1R for supporting most-if not-all cancer hallmarks associated with melanoma (78–85). However, the results from clinical trials targeting IGF-1R are still disappointing, emphasizing that we can learn to better assist the rational development of tyrosine kinase inhibitors for clinical use and which deserves a separate section (see below).

##### Drugs Targeting Signal Transduction Pathways

The last decade has seen significant advances in our understanding of melanoma biology, with the signaling pathway RAS/RAF/MEK/ERK being first to emerge as having a critical role. The RAS–RAF–MEK–ERK (also known as MAPK pathway) and the PI3K/AKT pathways are frequently activated in melanoma tumors (9, 86–90). Both pathways can be physiologically activated through RTK such as insulin-like growth factor receptors (IGF-1R), epidermal growth factor (EGFR), or c-Kit. The RAS proteins belong to a family of GTPases (including NRAS, HRAS, and KRAS) located on the inside of the cell membrane (89, 90). In melanoma, activating NRAS mutations (mainly at codon 61 and more rarely on codon 12–13) are found in approximately 20% of tumors (91).

Activating mutations in NRAS can cause parallel activation of both the RAS–RAF–MEK–ERK and the PI3K–AKT pathways (86, 89, 90, 92). RAS proteins phosphorylate and activate proteins of the RAF family of serine/threonine kinases (including BRAF and CRAF). In melanoma, the MAPK pathway is hyper-activated in up to 75% of cases, primarily due to somatic-gain-of function mutations in BRAF (~50% of cases) or RAS (~25% of cases). Activating BRAF mutations (mainly at codon 600) are found in approximately 50% of tumors, most often at V600E, V600K, or V600R (86, 89, 90, 92). The second most frequent BRAF mutation target the K601 residue. Both BRAF V600 and K601 hot-spot mutations are usually mutually exclusive with hot-spot NRAS mutations. In contrast, BRAF non-hot-spot mutations (including eight exon, 11 mutations) co-occurred with RAS (N/H/K) hot-spot and NF1 mutations (38).

Thus, there is an ongoing effort to develop small molecule inhibitors to target the B-RAF/RAS/MAPK pathway. Several B-RAF and MEK inhibitors are currently used both in current practice and clinical trials and more encouraging results from several clinical trials with new B-RAF inhibitors are being reported.

Yet, for instance, treatment with PLX4032, a BRAF inhibitor, leads to PFS of 7 months in patients with hyper-activated BRAF melanomas. Nevertheless, most patients who initially responded to treatment with PLX4032 relapsed, suggesting that on-going treatment with BRAF inhibitors is associated with development of drug resistance (93). The same initial response followed by resistance was also observed in outside and within other clinical trials targeting the RAS/RAF/MAPK pathway in melanoma, regardless of the drug used (94). Better results were, however, achieved by combination of two diverse inhibitors acting on different levels of the pathway (such as combined BRAF and MEK inhibition for instance). However, even in this case resistance has been described.

PTEN, the most frequently mutated gene in melanoma after BRAF and NRAS, promotes cell survival through sustained activation of the PI3K signaling pathways (95). Pictilisib is an oral selective PI3K-inhibitor that is currently being evaluated in clinical studies on multiple tumor types, including melanoma (96).

##### Nuclear Targets

The CDKN2A gene is frequently mutated or deleted in melanoma tumors, leading to a release in the inhibition of cyclin-dependent kinases CDK4 and CDK6, causing a progression from G1 to S-phase and increased cell cycle activity and proliferation (97, 98). Since the CDKN2A–CDK4/CDK6–cyclin D axis is often disrupted in melanoma CDK4/CDK6 inhibitors (e.g., abemaciclib and palbociclib) are predicted to be effective in the treatment of melanoma (99, 100).

The transcription factor MITF has a central role in the regulatory functions of melanocytes. MITF is regulated both through pigmentation pathways and Ras–Raf–MEK–ERK signaling (101). MITF regulates various cell processes through the transcription of proteins involved in pigmentation (TYR, TYRP1, and MC1R), proliferation and cell cycle regulation (p16, p21, CDK2) and apoptosis (BCL2) (102). MITF is considered to be an oncogene in melanoma as this gene locus is infrequently duplicated (103).

Other critical events in melanoma progression are mutations or deletions of the BRCA1-associated protein (BAP1) gene locus 148. The BAP1 gene maps to chromosome 3p21 and is a tumor suppressor gene that encodes a deubiquitinating enzyme (DUB) (104). As a deubiquitinase, BAP1 removes ubiquitin chains from its main substrate, histone H2A, thus controlling chromatin remodeling. BAP1 forms multiprotein complexes with several chromatin-associated proteins, including BRCA1, BARD1, and HCF1 (105). The normal BAP1 protein controls essential cellular pathways such as cell cycle control, cellular differentiation, transcription, and the DNA damage response. When lost due to inactivating mutations, germline BAP1 mutations cause a cancer syndrome whose characteristics are early onset of atypical Spitz tumors and increased risk of uveal and CM, mesothelioma, renal cell carcinoma, and various other malignancies (104). The exact role of BAP1 has not been entirely elucidated, earlier reports suggesting that the tumor suppressor function is linked to deubiquitinating BRCA1; however, later studies have indicated that BAP1 is a BRCA1-independent tumor suppressor (106). BAP1 alterations in UMs are often accompanied by somatic complete or partial loss of chromosome 3. This is consistent with a two hit model with activation of GNAQ/GNA11 and loss of activity of the tumor suppressor gene BAP1 (107, 108). As described earlier, telomerase function is also frequently defective in melanoma and drugs targeting such aberrations are likely to emerge in the near future.

Histone deacetylases (HDACs) are critically involved in epigenetic gene regulation through alterations of the chromatin status of DNA. Aberrant expression, dysregulation of their enzymatic activity or imbalances between HDACs and histone acetyltransferases are likely involved in the development and progression of cancer. Pharmacologic inhibition of HDACs show potent antitumor activity in a panel of malignancies, including melanoma. Trials are ongoing with several HDAC inhibitors, including entinostat (NCT02697630) (109).

##### Immune Targets

One of the fundamental roles of the immune system is to distinguish self from non-self. In the late 1950s, the hypothesis of cancer immune surveillance emerged, suggesting that cancers can provoke an effective immunological reaction with regression of the tumor (110). This is illustrated in immunocompromised patients who have an increased risk of cancers, including melanoma (111). Immune surveillance has been defined as having three key components: elimination (tumor eradication after antigen recognition), equilibrium (maintenance of tumor stability by immune control), and escape (tumor growth) (112). There are several clinical examples of melanomas being immunogenic tumors (113, 114). Another example of tumor elimination is metastatic melanoma with unknown primary, sometimes with a patch of vitiligo at the postulated site of the original lesion that may represent immune recognition and elimination of the primary melanoma, supporting this hypothesis (115). Late recurrences of distant metastases (sometimes after decades) in patients with early stage melanoma suggest prolonged periods of tumor equilibrium where tumor cells remain in a regional lymph node or at a distant site until a further event allows the tumor to escape.

The mechanisms underlying increased immunogenicity of melanoma compared to many other tumors are unclear. One hypothesis relates to the high mutation rates seen within melanomas compared with other tumor types. While cells with low mutational burden will display mostly normal cellular protein antigens on their MHC surface molecules without any immune activation, melanoma cells will display mutant proteins (tumor antigens), initiating an activation of the immune system (116, 117). Tumors often create an immunosuppressive microenvironment by mechanisms that prevent effective antitumor immunity. After antigen presentation, the surface CTLA-4 protein regulates and inhibits T-cell activation and suppresses the immune response. Another immune regulatory pathway involves the PD-L1 and PD-L2 ligands that are expressed on tumors and other cells and bind to PD-1 receptors on T-cells and inhibit their activation (118).

Among treatments tested in the last years are the immune checkpoint modulators, adoptive cell transfer like CARs, T-cell receptors (TCRs), and tumor-infiltrating lymphocytes (TILs), therapeutic antibodies and cancer treatment vaccines. Chimeric antigen receptor T (CAR-T) cell therapy is a newly developed adoptive antitumor treatment. Theoretically, CAR-T cells can specifically localize and eliminate tumor cells by interacting with the tumor-associated antigens (TAAs) expressing on tumor cell surface. Current studies (119) demonstrated that various TAAs could act as target antigens for CAR-T cells, for instance, the type III variant epidermal growth factor receptor (EGFRvIII) was considered as an ideal target for its aberrant expression on the cell surface of several tumor types. The third generation of CAR-T cell therapy demonstrates increased antitumor cytotoxicity and persistence through modification of CAR structure.

The adoptive transfer of *in vitro* cultured melanoma-reactive T cells isolated from autologous TILs after lymphodepleting chemotherapy has recently been shown to mediate objective tumor regression in 49–72% of patients with metastatic melanoma (120). However melanoma-reactive TILs could be generated from only 50% of resected samples (121). As a consequence, autologous T cells have been genetically engineered to express TCRs directed against shared tumor antigens like MART-1 melanocyte differentiation antigen or NY-ESO-1 (human tumor antigen of the cancer/testis family), highly expressed in many poor-prognosis melanomas (122).

The immune checkpoint inhibitors can act on the priming phase when the dendritic cell presents the information to the T cell by blocking the inhibitory signal *via* CTL4 or in the effector phase when the T cell acts on the melanoma cell by blocking the negative regulation *via* PD-L1. Additional research is aimed at understanding why checkpoint inhibitors are effective in some patients but not in others and identifying ways to expand the use of checkpoint inhibitors to other cancer types. In TIL therapy, Tumor Infiltrating Lymphocytes are harvested from the patient, expanded, activated with cytokines, and then reinfused into the patient. Ongoing clinical trials suggest that TIL therapy could be combined with PD-1 blockade agents as a first-line treatment or used in subsequent lines of therapy for patients with progressive disease (123).

Programmed death-ligand 1 is a 40 kDa type 1 transmembrane protein that has been shown to play a major role in suppressing the immune system by binding to PD-1 or B7.1. This event will transmit an inhibitory signal that reduces the proliferation of antigen-specific CD8+ T cells and/or CD4+ helper cells. Expression of PD-L1 in pretreatment tumor biopsy samples of melanoma correlates with response, PFS, and overall survival (124).

SD-101 is a registered investigational oligonucleotide with immune stimulatory CpG motifs that activates plasmacytoid dendritic cells *via* the toll-like receptor 9 (TLR9). By targeting TLR9 in both the early and late endosomes of plasmacytoid dendritic cells, SD-101 is intended to stimulate multiple mechanisms of tumor killing and to elicit a potent and focused innate and adaptive immune response to cancer. SD-101 was designed for highly efficient activation of the two principal TLR9 signaling pathways. One pathway leads to rapid Interferon-α production, which in turn stimulates several critical activities including activating natural killer cells, blocking immune suppression, and promoting Th1 and CD8+ T cell homing to the tumor. The second pathway leads to efficient generation and activation of tumor-specific cytotoxic CD8+ T cells (125).

##### Combination Treatments

Several clinical trials assessing combination of BRAF or MEK inhibitors with immunotherapy have been conducted or are ongoing (126). An initial phase I study where treatment-naïve patients received concurrent vemurafenib and ipilimumab was stopped after patients experienced severe hepatic toxicity. However, a phase I study with vemurafenib in combination with atezolizumab, an anti-programmed cell death-ligand 1 (PD-L1) agent, was more successful. Further, promising results regarding responses and tolerability have been reported in studies that are still ongoing with both the triple combination of dabrafenib, trametinib, and the PD-L1 inhibitor durvalumab (MEDI4736) and dabrafenib, trametinib, and the PD-1 inhibitor PDR001 (COMBI-i).

Retrospective analyses provide evidence that the effect of immunotherapy is reduced when given after progression on BRAF inhibitors. Therefore, clinical trials have been designed to help identify the optimal sequencing of combination therapies with BRAF/MEK inhibitors and immunotherapy. One trial (NCT02224781) compares dabrafenib/trametinib followed by ipilimumab/nivolumab versus ipilimumab/nivolumab followed by dabrafenib/trametinib. Another trial, the prospective three-arm randomized phase II SECOMBIT study (NCT02631447) compares a sequential approach with ipilimumab/nivolumab followed by encorafenib/binimetinib or *vice versa*. A third arm will involve an initial “pulse” of encorafenib/binimetinib for 8 weeks followed by ipilimumab/nivolumab until disease progression then again encorafenib/binimetinib until disease progression (126).

Several studies are ongoing with inhibitors of other immune costimulatory molecules such as IDO, OX40, CD137, and GITR (127). Of these, the IDO inhibitor epacadostat has come furthest with an ongoing phase III clinical trial where the drug is combined with pembrolizumab for metastatic melanoma (NCT02752074).

Preliminary results from a safety, tolerability, and dose escalation ongoing phase Ib/II study involving intratumoral SD-101 and pembrolizumab (Keytruda) have demonstrated that the combination was well-tolerated with no dose-limiting toxicities, according to Antoni Ribas, MD, Ph.D., who presented the findings at the 2016 Society for Melanoma Research (SMR) Annual Congress in Boston. The trial is conducted across 53 cancer centers in USA, Australia, and Europe and is still recruiting according to https://Clinicaltrials.gov (128).

Researchers reported an elevation of immune function, which was observed by increases in immune function signals, as well as increases in immune cell infiltrates, in the tumor microenvironment. Preliminary reported results included 18 patients with stage IIIc or IV melanoma. Eight patients were anti-PD-1 naïve, and 11 had previously received an anti-PD-1 therapy. The median follow-up for the anti-PD-1 naïve patients was 188 days, and the median follow-up for patients who had had anti-PD-1 was 81 days. Biopsies and CT scans were taken at baseline and throughout the treatment phase. SD-101 was given weekly for 4 weeks, and then given every 3 weeks coinciding with the doses of pembrolizumab. At the time of follow-up in the anti-PD-1 naïve group, 20% (*n* = 1) had had a CR, 60% (*n* = 3) had had a partial response (PR), 20% (*n* = 1) had progressive disease, and 80% (*n* = 4) had a PR or CR. For the experienced group, 50% (*n* = 4) had stable disease (SD) and 50% (*n* = 4) had progressive disease.

## Emerging Melanoma Therapies and the Future of Personalized Medicine

We are witnessing significant advances in our understanding of melanoma biology, with the signaling pathway RAS/RAF/MEK/ERK emerging as having a critical role. Thus, there is justification to develop small molecule inhibitors to target this pathway (129). For instance, in the case of treatment with PLX4032, reported data indicate a PFS of 7 months in patients with hyper-activated B-RAF melanomas. However, most patients who initially responded to treatment relapsed, suggesting that on-going treatment with B-RAF inhibitors is associated with development of drug resistance. The same initial response followed by resistance was also observed in other clinical trials targeting the RAS/RAF/MAPK pathway in melanoma. The corollary of these studies is that repressing B-RAF (or RAS) in melanoma cells triggers an alternative-signaling program, involving a switch, which allows the tumor to continue to rely on MAPK for maintenance of the malignant phenotype and drug resistance. RAS is a small G protein that is attached to the inner leaflet of the plasma membrane, RAF, MEK and ERK are cytosolic protein kinases whereas their effectors, e.g., activated ERK1/2 are nuclear transcription factors. In normal melanocytes, the MAPK pathway is activated by GPCRs and growth factors such as insulin-like growth factor 1 (IGF-1), SCF, fibroblast growth factor, and hepatocyte growth factor. In melanoma cells, the MAPK pathway is additionally sustained by oncogenic RAS or B-RAF mutations.

Among MAPK activators, IGF-1R is considered one of the most attractive targets for melanoma therapy. IGF-1R is responsible for the transformation, proliferation, and metastasis of melanoma and maintains the malignant phenotype (130–132). Moreover, others and we have clearly demonstrated that clinical inhibition of IGF-1R would be beneficial for melanoma treatment (81, 133, 134). Furthermore, *in vivo* and *in vitro* studies using IGF-1R antibodies, small molecule inhibitors, and antisense technology have shown that IGF-1R is functionally essential for melanoma cell growth and proliferation (78, 79, 81, 89, 90). IGF-1R signaling interfered with several crucial mechanisms involved in melanoma metastasis (135–137). IGF-1R inhibition reduced cell migration, invasion into basement membrane and endothelial cell barrier by decreasing cellular binding to several extracellular matrix proteins and blocking activity of matrix metalloproteinase 2. Most importantly, using animal models, we demonstrated that IGF-1R inhibition not only caused complete regression of melanoma xenografts but also drastically reduced the incidence of liver metastasis *in vivo*. However, it is crucial to identify the subset of patients likely to respond. While the strong preclinical evidence that suggests a clear benefit from inhibiting IGF-1R, the results of clinical trials using IGF-1R inhibitors indicate that tumors initially addicted to IGF-1R signaling develop rapid resistance to the therapy (131, 132, 138).

As explained before, the clinical success of nearly all tyrosine-kinase inhibitors is predicted by the presence of activating mutations or substantial receptor overexpression, but neither is the case with IGF-1R. As IGF-1R does not show intrinsic receptor abnormalities, it is likely that other pathways and quantitative changes are responsible. IGF-1R is classified as a RTK and accordingly tyrosine phosphorylation was considered to be the central process governing IGF-1R signaling (139). However, during the last decade, we challenged this view by demonstrating the involvement of ubiquitination in IGF-1R function (80, 140). We demonstrated that β-arrestin1 (β-arr1) serves as an adaptor to bring the E3 ubiquitin-ligase Mdm2 to the IGF-1R, with a dual outcome on IGF-1R: ubiquitination and receptor downregulation as well as IGF-1R/β-arr1 mediated activation of the MAPK signaling (82, 141). It should be highlighted here that β-arrestins have emerged as remarkably versatile adaptor molecules well known as coordinators of signaling pathways downstream GPCRs. While shutting down the G proteins-signaling β-arrestins redirect the signaling to MAPK. As such, the dual regulatory role of β-arrestin 1 in the case of IGF-1R is remarkably similar to the role played by the β-arrestins in the case of the GPCR family: while downregulating IGF-1R, β-arrestins redirect the signaling to MAPK (131, 132). Building on this observation, we investigated the β-arrestin-IGF-1R binding mechanism and revealed the missing link that would functionally portray a prototypical RTK, the IGF-1R, as a GPCR (142): GRK-dependent phosphorylation of IGF-1R serine residues as the underlying mechanism for β-arrestin binding. Finally, we confirmed the functional consequences of GRK regulation of β-arrestin recruitment on IGF-1R trafficking and signaling and found that GRK6 targets the receptor for degradation, whereas GRK2 mediated phosphorylation results in receptor endocytosis followed by recycling to the plasma membrane (142). Recently, we specifically investigated the role of the other β-arrestin isoform (β-arr2) and revealed that the two β-arrestin isoforms antagonize each other’s functions at the IGF-1R (89, 90). Strategies to imbalance toward the β-arrestin 1 isoform (β-arr1 plasmid overexpression or β-arr2 siRNA silencing), increase ligand dependent receptor degradation, and β-arr1 mediated MAPK signaling. β-arrestin 2 acts to enhance receptor degradation in the absence of the receptor and protects the receptor against ligand-dependent degradation, and counteracts against β-arrestin 1 signaling. This study also revealed an additional cancer relevant scenario—while β-arrestin 1 signaling downstream of IGF-1R acts to inhibit p53, removal of this through β-arrestin 2 allows for p53 accumulation (89, 90). This study suggests that strategies to imbalance β-arrestin isoform expression toward β-arre2 downstream of the IGF-1R limit MAPK activation while reactivating a tumor suppressor, a strong double-hit system for therapeutic targeting in cancer cells. This is of paramount importance as melanoma tumors usually retain wild-type p53; however, its tumor-suppressor activity is functionally disabled, most commonly through an inactivating interaction with mouse double-minute 2 homolog (Mdm2), indicating p53 release from this complex as a potential therapeutic approach. P53 and the tumor-promoter IGF-1R compete as substrates for the E3 ubiquitin ligase Mdm2, making their relative abundance intricately linked. Considering that specific targeting of one signaling module creates a selective pressure within an already unstable system, we were investigating the effects of directing the Mdm2-ligase toward IGF-1R. Small-molecule inhibitors have been developed with the aim of reactivating p53 by preventing its interaction with Mdm2. Nutlin-3 is one such small-molecule inhibitor, which binds to Mdm2 in the p53-binding pocket, thus preventing Mdm2-mediated p53 degradation, stabilizing p53, and additionally increasing its synthesis rate causing growth arrest. By pharmacologically releasing Mdm2 from the Mdm2/p53 using Nutlin, one can increase IGF-1R/Mdm2 association with enhanced IGF-1R ubiquitination, receptor downregulation and selective downstream signaling activation confined to the MAPK pathway. IGF-1R downregulation synergizes with MEK1/2 inhibition, by removing a crucial back-up pathway available to melanoma cells. Our data clearly demonstrate that Nutlin-3 treatment completely inhibited the invasiveness of melanoma cells through an impaired IGF-1-mediated matrix metalloproteinases type 2 activation mechanism (92). Hence, Nutlin-3 can destabilize p53/Mdm2/IGF-1R circuitry and this could be developed for therapeutic gain.

The appreciation of the dual functions of β-arrestin, as a mediator of IGF-1R signaling as well as mediator of receptor downregulation provide the basis for the emerging paradigm of IGF-1R signaling as a hybrid RTK/GPCR. In this model, IGF-1R can initiate classical kinase signaling, β-arrestin signaling, and heterotrimeric G-protein signaling as well as β-arrestin-mediated receptor desensitization. However, the receptor conformation activating the kinase signaling can be distinct from that which interacts with β-arrestins, as demonstrated by the IGF-1R mutants constitutively binding β-arrestin, that are degraded even in the absence of the ligand. In this emerging model, not all receptors are equal and their activity can be modulated from inside the cell by particular posttranslational modifications (e.g., serine phosphorylation, ubiquitination, etc.) or by interacting proteins (e.g., β-arrestins, integrins, other RTKs) (86, 143, 144). These IGF-1R imbalances were explored for their ability to compensate for signals lost following therapeutic MAPK-inhibition. IGF-1R conformational changes associated with its inhibition can preferentially activate MAPK-pathway in a kinase-independent manner, a process known as biased signaling (131, 132, 144, 145) thus limiting the efficacy of MAPK inhibition. In a panel of skin melanoma cell lines with differing MAPK and p53 mutation status, specific siRNA toward IGF-1R downregulates the receptor and all its signaling in a balanced manner, while IGF-1R targeting by small molecule Nutlin-3 parallels receptor degradation with a transient biased pERK1/2 activity, with both strategies synergizing with MEK1/2 inhibition (89, 90). IGF-1R downregulation by a targeted antibody (Figitumumab) induces a biased receptor conformation that sustains MAPK activity and competes with the MEK1/2 inhibition. Our results indicate that IGF-1R downregulation offers an approach to increase the sensitivity of melanoma cells to MAPK inhibition, while highlighting that a good understanding of the molecular mechanisms could provide greater specificity and precision required for multi-hit personalized therapy (89, 90).

For all the abovementioned reasons, we can consider the β-arrestin system as the major switch machinery for MAPK signaling, supporting its continuous activation by employing components of GPCRs, RTKs, or both while also controlling p53 expression and activation.

## Conclusion

We have witnessed a dramatic change in the management of melanoma in the last decade with significant improvements in patient outcomes and a shift toward more patient centered treatments. Personalized medicine, based on specific markers and mutations, is a rapidly growing field and the increased knowledge of molecular targets and drugs tailored accordingly will undoubtedly continue to move the field of melanoma treatments further forward.

## Author Contributions

AG conceived and planned the paper; AG wrote the manuscript with contribution from HH and ID. HH contributed to the outline of the paper.

## Conflict of Interest Statement

The authors declare that the research was conducted in the absence of any commercial or financial relationships that could be construed as a potential conflict of interest.
